# Moles, Warts, Etc.

**Published:** 1878-12

**Authors:** 


### *MOLES, WARTS, ETC.*

Although perfectly innocuous, these excrescenses are always unsightly, and the physician is frequently applied to to adopt measures for their removal. WARTS may exist for years and then disappear, without treatment; or their exit may be hastened by some simple domestic remedy. Not so, however, with MOLES. If left to themselves they remain during life, frequently to annoy by their unfavorable location, which causes them to be often painfully wounded. Besides, they are not ornamental, and the desire to be rid of them can hardly be called a weakness. We dress and adorn our persons to make ourselves more agreeable to others, and to increase our self-respect, and there is no reason why we should not divest ourselves of those things which are to a greater or less extent drawbacks to our efforts in this direction. The removal of Moles is easy of accomplishment, if that is the sole object sought. But their proper removal is quite another matter, and requires skill and experience, beside a thorough anatomical knowledge of the parts operated upon. A physician possessing these requirements will quickly and safely remove a Mole, so that in two days no traces of its existence can be seen. The scars so frequently left after the removal of Moles by the painful process of destroying them with acids, are a greater deformity than the Moles themselves, and render a second operation for their removal desirable. Either of these operations, when carefully performed, is entirely painless, and most satisfactory in its results. Unsightly scars from other causes, if not too extended, can be improved in appearance, and sometimes completely obliterated, by an operation that causes no pain and but little inconvenience to the patient. All operations of this kind relate to "plastic surgery." While not essential to the saving of life, they are great conservators of good looks, which, with some people, seems quite as essential.

### MOTH PATCHES.

These are another source of annoyance, particularly to women, who have almost a monopoly of them. Their removal is quickly accomplished, in the great majority of cases, where local treatment only is necessary.

### ACNE OR PIMPLES

are generally supposed to indicate good health ; but it is certain that the most robust health can be supported without their offensive presence. Beside, their existence for a long time produces pits which permanently disfigure the face. They should be judiciously treated and promptly cured. They yield readily to treatment.

### NÆVUS OR BIRTH-MARK.

While Moles, Moth Patches, and eruptions on the face yield promptly to proper treatment, Birth-marks present, in some cases, obstacles that it is very difficult to overcome. The former affections are confined entirely to the skin, while Birth-marks frequently extend into the tissues beneath the skin, and are involved with nerves, veins, and arteries. Treatment will often effect cures, and will, even in the worst cases, be of benefit. The physician should proceed cautiously, after studying each case as it is presented. We are constantly treating such cases.

